# Combination of Micronutrients for Bone (COMB) Study: Bone Density after Micronutrient Intervention

**DOI:** 10.1155/2012/354151

**Published:** 2012-01-15

**Authors:** Stephen J. Genuis, Thomas P. Bouchard

**Affiliations:** ^1^Faculty of Medicine, University of Alberta, Edmonton, AB, Canada T6K 4C1; ^2^Department of Family Medicine, University of Calgary, Calgary, AB, Canada T2N 1N4

## Abstract

Along with other investigations, patients presenting to an environmental health clinic with various chronic conditions were assessed for bone health status. Individuals with compromised bone strength were educated about skeletal health issues and provided with therapeutic options for potential amelioration of their bone health. Patients who declined pharmacotherapy or who previously experienced failure of drug treatment were offered other options including supplemental micronutrients identified in the medical literature as sometimes having a positive impact on bone mineral density (BMD). After 12 months of consecutive supplemental micronutrient therapy with a combination that included vitamin D_3_, vitamin K_2_, strontium, magnesium and docosahexaenoic acid (DHA), repeat bone densitometry was performed. The results were analyzed in a group of compliant patients and demonstrate improved BMD in patients classified with normal, osteopenic and osteoporotic bone density. According to the results, this combined micronutrient supplementation regimen appears to be at least as effective as bisphosphonates or strontium ranelate in raising BMD levels in hip, spine, and femoral neck sites. No fractures occurred in the group taking the micronutrient protocol. This micronutrient regimen also appears to show efficacy in individuals where bisphosphonate therapy was previously unsuccessful in maintaining or raising BMD. Prospective clinical trials are required to confirm efficacy.

## 1. Introduction

Disordered bone health is an age-related illness that affects an increasing proportion of the population in many western nations. Throughout much of the developed world, the fastest growing segment of the current population is the baby-boomer generation, the group born during the post-WWII baby boom that is rapidly approaching retirement. According to the Statistics Canada 2006 Census, for example, baby-boomers account for one-third of the country's 32 million people, 20% of which are in the 55–64 age class and soon to leave the workforce [1]. Older patients with low bone density are at high risk for falls and fragility fractures [2], which in turn cause considerable morbidity and subsequent mortality as well as exerting an enormous financial burden on public health care systems [3]. With an aging population, prevention of age-related diseases including osteoporosis and related fragility fractures will continue to play an important role in the sustainability and implementation of good personal and public health care.

As improved bone mineral density (BMD) has been associated with a diminished risk of fragility fractures, preferred BMD status in greater proportions of the population would deliver not only improved quality of life but also significant cost savings. With escalating rates of osteoporosis in various jurisdictions over the last decade, it would be desirable if primary prevention strategies to obviate the development of compromised bone health could be instituted as well as nontoxic interventions to restore bone strength in those with deficient BMD.

Recent clinical practice guidelines for the diagnosis and management of impaired bone health have primarily focused on pharmacologic therapy and lifestyle modifications to prevent fragility fractures and their adverse sequelae [4–6]. Pharmaceutical interventions to address abnormal bone density have focused to a great degree on antiresorptive bisphosphonates. Other medications considered in select situations may include other antiresorptive agents such as a human monoclonal antibody RANK ligand inhibitor (Denosumab), a bone forming analog to parathyroid hormone (Teriparatide), strontium ranelate, calcitonin, and hormonal replacement therapy or a selective estrogen receptor modulator for postmenopausal women [4, 7].

A recent study has shown, however, that lack of compliance with current osteoporosis protocols is putting the elderly at increased risk for fragility fractures and associated morbidity and mortality [8]. Moreover, increasing numbers of patients decline osteoporosis pharmacotherapy because of media attention to potential adverse effects and legal proceedings related to outcomes alleged to be connected with some osteoporosis medications. For example, recent concerns about long-term hormone replacement therapy [9, 10] and media reports about atypical fractures [11], osteonecrosis [12], atrial fibrillation [13], and esophageal cancer [14] allegedly associated with bisphosphonate use has led some patients to pursue other approaches to ameliorate bone health despite the fact that the link between these medications and all the purported adverse side effects still remains controversial [13, 15].

Various micronutrients have recently been identified in the scientific and biochemistry literature as integral to the proper development, physiology, and maintenance of bone. Depletion of essential nutrients for bone health because of inadequate intake, impaired digestion, malabsorption, or disordered assimilation may result in deficient biochemistry, disordered biology, and resultant bone health compromise. Thus far, however, assessment and maintenance of nutritional adequacy in relation to the spectrum of essential compounds required for proper bone function has been limited, as micronutrient strategies have focused almost exclusively on calcium and vitamin D supplementation [4]. Recent guidelines have not yet incorporated the fact that some patients with osteoporosis may be malnourished in relation to other essential bone nutrients.

Recent research suggests that remediation of nutritional insufficiency and repletion of various biochemicals integral to healthy bone physiology may ameliorate bone health status [16, 17]. This retrospective cohort study, approved by the Health Ethics Research Board at the University of Alberta, assesses the value of the use of a combination of micronutrients on BMD status.

## 2. Methods

A review of the medical and scientific literature was undertaken to identify micronutrient elements associated with bone health status [16] by assessing available medical and scientific literature from MEDLINE/PubMed, as well as by reviewing numerous books, nutrition journals, and health periodicals, conference proceedings, and government publications. References cited in identified publications were also examined for additional relevant writings. The evidence base to support the role of specific essential micronutrients in bone status ranges from scant to very firm, depending on the compound [18]. Multiple studies have demonstrated that micronutrients (and drugs derived from nutrients) beyond just calcium and vitamin D have an impact on bone health. Vitamin K_2_ [19, 20], strontium [16, 21–25], magnesium [26], and DHA [27–30] have all been implicated in improving the status of bones, but to our knowledge, none of these individual micronutrients have been assessed when given in combination.

The first author practices environmental medicine, where many referred patients present with long-term chronic disease. With the view that comprehensive fracture risk assessment should be a routine part of patient care, and the observation that patients with chronic disease have higher rates of bone compromise and frequently do not receive therapy to prevent fractures [31–33], bone health determination was established as a component of the overall clinical assessment in chronically ill patients. Starting in 2006, patients found to have suboptimal BMDs were provided with options for management, including micronutrient therapy. As well as discussion related to lifestyle and standard-of-care pharmaceutical interventions, the potential consequences of not intervening, and the scientific literature on the published efficacy of micronutrient interventions were presented to patients along with information on recommended clinical practice guidelines for compromised bone health.

Some patients adamantly refused to use pharmaceutical therapies while others reported they had previously discontinued such therapy because of continued loss of BMD. A portion of these individuals indicated interest in supplemental nutrients linked in the scientific literature to improved bone health status. As patients presenting to environmental health specialists often have chemical sensitivities [34], the reluctance expressed to using pharmaceuticals was sometimes related to a sensitivity to medications or excipients commonly used within dispensed drugs.

Patients wishing to explore micronutrient use were given medical literature detailing the benefits purported in various studies. Eager to ameliorate their bone health status if possible, some individuals chose to use micronutrients rather than using pharmacologic therapies or not using any intervention at all. After 12 months of consecutive micronutrient therapy, repeat BMDs were performed to assess for evidence of change. A retrospective review of the outcomes was undertaken to gather data for analysis.

### 2.1. Demographics and Inclusion Criteria

The population of patients for the study came from an environmental medicine clinic in Edmonton, Alberta, Canada. Out of 219 patients assessed, 16 were still in the process of taking supplements (data not yet complete) and 126 were excluded. Exclusion criteria included patients with recognized bone compromising medical conditions or those on medications known to potentially affect bone health. (e.g., 1 with anorexia nervosa and 1 on chemotherapy) as well as patients who had repeat BMD measurements inadvertently performed on different machines from the original (5 patients)—making comparison inaccurate. In addition, 37 patients were excluded because they did not comply with therapy (the micronutrients were taken inconsistently—self-reported at less than half the time), 6 patients commencing the protocol changed their mind about taking the nutrients (for financial reasons), and 71 patients did not return at the end of the time period for follow-up BMD assessment or decided they did not want a repeat BMD and therefore had incomplete data. Two patients died during the course of the study, unrelated to the intervention (1 from ALS and the other from a motor vehicle accident). The final sample of 77 patients taking the micronutrient combination most or all of the time was included with complete data for analysis.  Table 2 shows the demographics for the study group.

### 2.2. Protocol and Rationale

Each patient in the analysis who chose to use the micronutrient intervention followed a suggested daily protocol of supplemental nutrient consumption and exercise as described in Table 1. It was hypothesized that perhaps bone compromise might be related to nutritional insufficiency in some patients and that remediation of nutritional biochemistry may be of assistance in restoring bone health. Also, both DHA and vitamin D are involved in genetic regulation of many genes and restoration of optimal levels has been associated with improved bone strength [35–38]. Given the debate about the efficacy of calcium supplementation for reducing fractures [39, 40] and the potential risks associated with high-dose supplementation including renal calculi and cardiovascular events [40, 41], patients were advised to obtain calcium from dietary sources including vegetables such as Brussels sprouts or broccoli rather than calcium supplements. Patients were also instructed to commence and maintain a regimen of daily impact exercises such as jumping jacks or skipping where possible as impact has been associated with prevention of bone density loss [42, 43].

As BMD is a major determinant among several risk factors for predicting fragility fractures, BMD follow-up measurement was therefore used as an intervention outcome along with fall surveillance. The main areas analyzed for bone density included the lumbar spine, the femoral neck, and the femoral trochanter. In this study, we evaluated comparative differences in the femoral neck, total hip, and total spine in relation to previous studies. We also investigated change at the lowest hip and lowest spine sites to determine whether there was improvement in the areas that were the least dense and potentially the most vulnerable.

### 2.3. Statistics

Statistics were calculated with SPSS 18.0 (IBM Corporation, USA). Pre- and postintervention bone densities were compared using a one-way ANOVA with a *P* value threshold of 0.05. Changes were also evaluated using mean percentage change over one year to compare treatment effect with the bisphosphonates and strontium ranelate. In the analyses, z-scores were used in order to avoid any age-related bias in some other BMD scores.

In addition, a post hoc analysis of the noncompliant group (*N* = 37) was carried out to evaluate whether adherence to the combined micronutrient strategy was beneficial. One-way ANOVA with a *P* value threshold of 0.05 was again used to determine if this group showed a significant difference over the course of the one year period. Moreover, the percentage change at each site in this group was compared to the percentage change in the intervention group.

Nineteen patients (25%) were classified in the normal category despite having low bone mass or suboptimal levels when age-matched to general population standards. According to the interpretation of the reports provided, the “bone quality in younger individuals differs from that of older people” and thus “absolute fracture risk has not been determined in this population.” As such, diminished bone health with levels considerably lower than the mean are still placed in a “normal” diagnostic category. BMD testing was done on various younger patients as many of these individuals presented with chronic illness and had evidence of irregularities on biochemical nutritional status testing, or had other factors that might predispose them to bone health compromise.

## 3. Results

The population included predominantly women (94%) who were mostly postmenopausal (81%). Of these patients, 29 (38%) reported lack of success with previous use of bisphosphonates and 48 (62%) declined standard drug therapy. The distribution of bone densities in the sample is summarized in Table 2.

After treatment, there was a significant improvement in bone density (z-scores) in the femoral neck, total spine as well as lowest hip and spine scores in the overall group (Table 3). Improvement was observed in the total hip scores, but this change was not significant.

**Figure 1:**
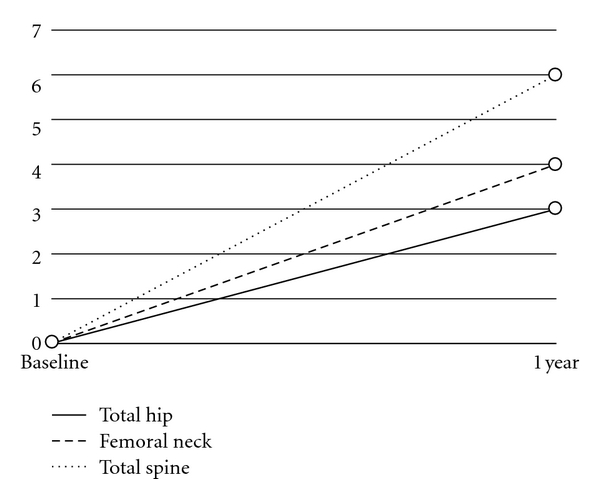
Mean percent change in bone density from baseline in the intervention group.

Overall percentage change after one year is presented in Figure 1 and comparisons with published results for selected pharmaceutical interventions are presented in Table 4. The percent changes in the entire group as shown in Table 4 were the same when the whole group (males and females, all ages) was analyzed as when only postmenopausal females were analyzed. Isolating only the postmenopausal females with osteopenia and osteoporosis also revealed the same percentage changes with the exception of the lowest hip site improving 5% with this subgroup rather than 4% for the overall group. In the five males, there was an even greater percentage change: 10% in the femoral neck, 8% in the total hip, 10% in the lowest hip site, 10% in the total spine, and 16% in the lowest spine site. Table 4 compares the results in the current sample of patients using combination of micronutrients to strontium ranelate alone [21], as well as to published bisphosphonate trials of Alendronate [44] and Risedronate [45].

**Figure 2:**
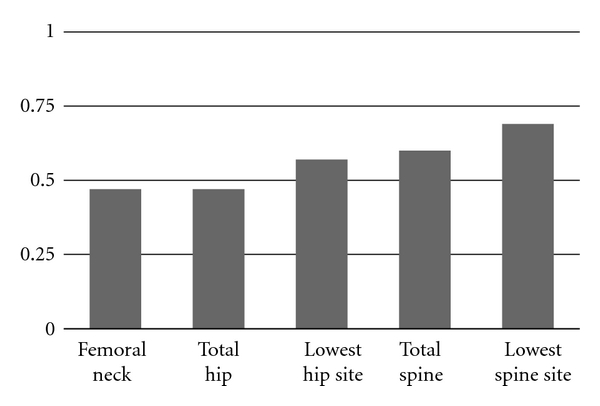
Proportion of patients showing >3% change in the various sites within the first year.

The BMD change in one year was more pronounced in the hip (femoral neck and lowest hip site) among those who reported lack of success with previous bisphosphonate therapy (Table 5) and more pronounced in the lowest spine site among those who had chosen to decline primary bisphosphonate therapy. Over the course of the study period, there were no fractures from ground level falls in any of the participants. Finally, Figure 2 summarizes the proportion of patients who experienced a BMD change of greater than 3% within the first year of following the COMB protocol, suggesting rapid onset of BMD improvement for many participants.

**Figure 3:**
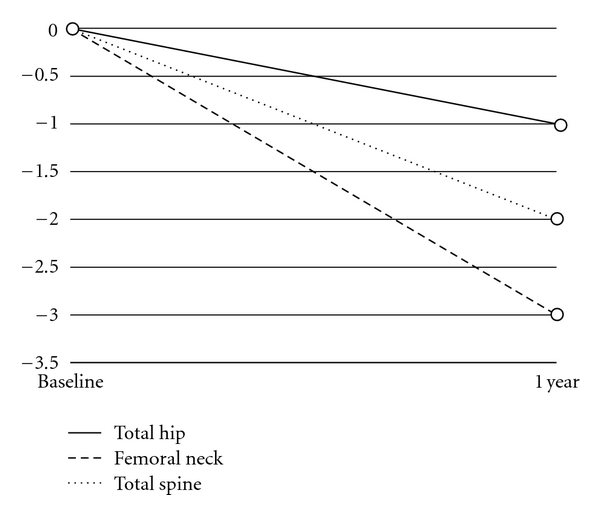
Mean percent change in bone density from baseline in noncompliant group (*N* = 37).

Compliance issues have become evident in this study. Many patients did not complete the 12 month course consistently, but took the intervention sporadically. In this group of patients (*N* = 37), a post hoc analysis was carried out to determine whether sporadic supplementation would be of benefit. There was no significant difference in z-scores at any of the sites after one year (*P* > 0.05). At one year, the percentage change in the femoral neck was −3%, the total hip was −1%, the total spine was −2%, the lowest hip site was −2%, and the lowest spine site was −1%.  Figure 3 shows the percentage change after one year in the noncompliant group (compared to Figure 1 in the compliant group).

## 4. Discussion

Diminished BMD is an important indicator of compromised bone health and has been established as a determinant associated with fragility fractures. Integrative approaches for preventing fragility fractures will be essential in addressing the health concerns in our aging baby boomer population. In selected patients with diminished bone health, combined micronutrient therapy may be a promising alternative to pharmaceutical strategies in order to prevent bone compromise as well as to maintain or to improve BMD. In this study, we observe that an expanded micronutrient combination alone can improve BMD in many patients who failed to achieve success with bisphosphonate medications as well as those who declined to start bisphosphonate therapy for reasons of choice or chemical sensitivity.

### 4.1. Limitations, Confounder, and Strengths

Limitations of this study include the small sample size and the lack of a blinded placebo-controlled group. Furthermore, given the multiple intervention nature of the combination micronutrient regimen, it is difficult to pinpoint which nutrients or nutrient groups were ultimately responsible for the improved BMD in each case. As well, the one-year followup limits the ability to determine the long-term effects of this regimen on BMD measurements and sustained prevention of fragility fractures.

There is also marked selection bias in this study group which might potentially lead to an underestimate of the full potential of these interventions in the general population. Some of these patients, for example, have unsuccessfully tried pharmacologic therapies for many years and thus represent a skewed portion of the population. Furthermore, many of these patients have multisystem health problems that may inhibit normal physical activity or may be associated with other pathophysiologic mechanisms impairing proper bone physiology.

Each micronutrient in the regimen has prior published data to suggest effectiveness in improving bone health, but this is the first study to our knowledge that examines these micronutrients in combination. While it is impossible to determine which component or components of the micronutrient combination were able to achieve the benefit realized, the issue of isolating the individual effective component(s) of the COMB protocol is more of a theoretical than practical concern. Biochemicals in their natural physiological state as produced in foods or gut microbiota do not work in isolation. Combinations of nutrients are known to be required for normal biochemical function. For example, both vitamin D and magnesium are required for proper calcium deposition and bone development [26]. In addition, emerging evidence suggests that other nutrients including some phytochemicals may contribute to the constellation of factors involved in healthy bone biochemistry [46]. Using single supplemental biochemicals in isolation may not be successful, whereas using them in combination may be efficacious.

The contention that a combination intervention is less credible, and that a traditional prospective clinical trial isolating individual variables to determine independent efficacy compared to controls is required to demonstrate benefit and to recommend micronutrient therapies, is debatable. With the emergence of molecular medicine and the Human Genome Project (HGP) recently identifying each person as biochemically unique, it may not be valid to say that any single therapy used broadly will work in a similar fashion for individual patients as genomic variability already introduces multivariables. The HGP has demonstrated that genomic solitary nucleotide polymorphisms (SNPs) regulate genomic function and affect the function of enzymes they code for, thus raising the question of what constitutes a proper control group [47, 48]. Most clinical trials to date have controls based on race, age, and sex, but genomic variables with SNP variability may be just as significant as race, sex, and age as determinants of physiological outcome. Emerging evidence shows that SNP variability is enormous within race and sex groups and many published clinical trials, including osteoporosis research, which omit relevant genomic information may not have proper controls and are, at best, anecdotal.

The rapidly emerging field of genomics is increasingly supplanting knowledge gleaned from broad-based clinical trials in many branches of medicine [49–51] and has ushered in the expansion of pharmacogenomics and nutrigenomics to specifically assess and treat individuals according to their unique biochemistry [52]. Furthermore, the Human Microbiome Project has recently uncovered the individual nature of the gut microflora [53], creating further evidence of individual biochemistry, a unique gut microbiome, and resultant unique physiology. Broad-based research without genomic controls, identifying a single pharmacologic agent as a widespread therapy for osteoporosis or any other condition may be judged to have inadequate experimental design and thus scientifically unreliable. Accordingly, reproducible clinical interventions which yield positive clinical outcomes, as in this study, definitely have limitations but may have at least comparable merit to traditional trials when considering benefit.

### 4.2. Strontium Citrate and Strontium Ranelate

It has repeatedly been documented in the literature that pharmacologic therapy with strontium ranelate is associated with an elevation in BMD as well as reduction in fragility fractures [16, 21–25]. Since strontium is a metal in the same group of periodic elements as calcium, it has been recognized that strontium in high concentrations may displace and replace calcium in bone by heteroionic exchange [54], a phenomenon which has elicited disparaging regard for strontium therapy among some bone specialists. Rather than an increased BMD, however, this physiochemical process in the presence of excessive strontium ultimately results in decreased bone calcium content [55], dissolution of mineralized bone [56], disruption of bone architecture [57], and lower BMD [58]. This phenomenon only appears to be the consequence of disproportionately high doses of strontium intake, not regular supplemental levels at low dose.

At low supplemental doses of strontium, in fact, there is evidence of an increase in both the bone formation rate and the trabecular bone density related to a strontium-induced stimulation of osteoblastic activity [58]. Furthermore, at low doses, strontium is not associated with any mineralization defect or any increase in the number of active bone-resorbing cells [59, 60]. In addition, it has recently been found that the mechanism of strontium benefit may also involve a calcium preservation effect as the rate of calcium release was almost halved after strontium treatment was assessed in recent research on teeth [61]. Finally, strontium supplementation, unlike use of calcium supplementation, shows ability to recalcify osteopenic areas in pathological bone conditions characterized by accelerated bone loss and extensive demineralization [58, 62].

Strontium is increasingly being recognized as a trace mineral which may be essential to the normal biology of bone and teeth and it is yet undetermined if strontium deficiency, like iodine deficiency, results in physiological malfunction [63]. It has been recently reported that commercial foods grown on fields using synthetic fertilizers, pesticides, and herbicides have appreciably lower levels of strontium than organic food counterparts [64]. Thus the restoration of adequate strontium levels to individuals may simply represent the normal homeostatic requirement for strontium, and normal healthy bone may require some level of strontium to prevent calcium loss [61]. Most importantly, treatment to elevate strontium levels has repeatedly been shown to demonstrate safe and remarkable efficacy at diminishing fractures in hip, vertebral as well as peripheral sites [21–25].

Studies to date have predominantly focused on strontium ranelate rather than the readily available strontium citrate supplement as used in this study. The results of this study, however, demonstrate that the micronutrient combination including strontium citrate is at least as effective in BMD change as strontium ranelate with suggestion of preferred efficacy of the former therapy at improving femoral neck outcomes. Furthermore, the ranelic acid salt is a purely synthetic molecular compound, while citrate is naturally occurring. It appears to be the strontium portion of the molecules which exerts most or all of the positive effect on bone. When consuming the strontium ranelate, for example, the compound splits into two strontium ions and one molecule of ranelic acid, with each absorbed separately. There is little evidence that the ranelic acid portion of the strontium ranelate compound contributes to the effect of strontium on skeletal tissue, and of the small amount of ranelic acid that is absorbed into the body, almost all is excreted within a week without ever being metabolized. All forms of strontium have bioavailabilities in the 25–30% range, but gastric tolerance appears to be better with the ranelate and citrate forms.

With the mounting concern about the safety profile of some standard medical interventions for bone compromise, strontium is very well tolerated and has shown remarkably little in the way of side effects or long-term adverse sequelae. An increased risk of thrombosis has been noted with strontium ranelate, an effect not reported (to our knowledge) with strontium citrate [16].

### 4.3. Mechanism of Action of Micronutrients

Unlike pharmacologic interventions, it is hypothesized that micronutrient strategies do not work by altering physiological parameters such as osteoclast function, but rather function by remediating underlying nutritional deficiencies which then permit restoration of inherent physiological processes. It is increasingly documented that nutritional deficiency continues to be an unrecognized and undertreated problem in clinical practice [65, 66]. Compromised nutritional status has recently been correlated with diminished quality of life and increased morbidity and mortality [67, 68]. It is well established that adequate weight and BMI, oft assumed to indicators of nutritional sufficiency, underestimate nutritional status and risk [69]. Investigation and management of malnutrition, often found in those with chronic disease, should become standard practice in clinical medicine [65, 70].

### 4.4. Relative Cost of COMB Protocol

An important factor to consider in evaluating this combination of micronutrients for bone health is the cost to patients, given that supplements are generally not covered by public formularies while many pharmaceuticals used for osteoporosis receive coverage. The current COMB protocol was evaluated at $2.26 (CDN) per day, amounting to $67.80 per month or $824.90 per year. Bisphosphonates, on the other hand, range from $0.90 (least expensive generic preparation) per day to $12.96 (brand name) per day for Risedronate and $1.10 per day (least expensive generic) to $5.58 per day (brand name) for Alendronate (according to Blue Cross coverage for Alberta, Canada). A small percentage of the group discontinued the micronutrient intervention because they felt it was too expensive to purchase the nutrients, which were not covered by their drug plans. Given the potential cost saving in maximizing bone health, it would be prudent for government formulary administrators to consider funding such a protocol in appropriate patients.

### 4.5. Public Health Considerations

From a public health perspective, a number of fundamental questions need to be addressed.

- Why is there an epidemic of impaired bone health?
- Why is the incidence and prevalence of osteoporosis increasing?
- Why is there disparity in the geographic distribution of osteoporosis?
- Why does osteoporosis frequently occur in individuals with no family history of bone compromise?

Genetics have not changed in the last three decades but lifestyle and environmental factors influencing bone health have. While use of pharmaceuticals may diminish risk of fracture in individual cases, they do not address the etiology or underlying cause of bone compromise; osteoporosis is not a bisphosphonate-deficiency disease. Accordingly, any prevention strategy must investigate and address lifestyle, nutritional and environmental determinants that have contributed to the rise in bone health compromise. The marked improvement in BMD with simple micronutrients in this study raises the question as to whether nutrient deficiency is a widespread phenomenon and a major determinant of this public health problem. Comprehensive research on nutritional status of patients with osteoporosis needs to be undertaken to determine if nutritional deficiency is a factor.

It has been well documented that vitamin D insufficiency is a widespread reality and a determinant of myriad health problems including bone compromise [71]. A challenge with the consideration of nutritional status assessment, however, is that levels of some essential nutrients for bone metabolism such as strontium and vitamin K_2_ are not yet available in most laboratories. Accordingly, clinical suspicion, laboratory testing where possible, and repletion of nutrients required for normal bone physiology may represent the best that can be done with regards to nutritional management at the current time.

## 5. Concluding Thoughts

Osteoporosis has become a serious personal health issue for countless individuals as well as a disturbing public health problem for many countries as it now affects up to 1 in 2 women and 1 in 5 men over the age of 50 in some population groups [5]. Fragility fractures associated with impaired bone health account for widespread morbidity and, in the case of hip and vertebral fractures, undue rates of mortality [4]. Public expenditures associated with the management of osteoporotic fractures and their complications are staggering [3]. Left untreated, impaired bone health often has debilitating sequelae for individuals and profound implications for public health care.

The current practice standard for making a diagnosis of impaired bone health involves bone density measurement in conjunction with determination of clinical risk factors. Based on this combined assessment, clinical decisions to intervene with treatment are routinely made. The objective of any treatment to improve bone health, medications or otherwise, is to reduce the risk of fragility fractures in the future. It has been repeatedly established that those individuals with deficient bone mineral density, as measured by densitometry testing, are at increased risk for fragility fractures [72, 73]. It has been found that timely and effective management of compromised bone health, as diagnosed in part by suboptimal BMD measurements, can reduce fracture risk [6]. Measures which are successful in improving BMD measurements have been found to diminish the risk of fragility fractures [21, 74].

Interventions to improve BMD usually include the use of bisphosphonate or other pharmacologic options including teriparatide, strontium ranelate, raloxifene, hormone therapy, or calcitonin. However, there are some individuals who do not tolerate these medications, some that have not experienced improved BMD with these treatments, and some who decline to take these therapies because of reluctance to use medication in general, or because of increasing media attention to potential adverse effects associated with some osteoporosis drugs. Accordingly, some authors have recommended that nonpharmacologic strategies to improve or maintain bone health be included in discussion of options for bone preservation and therapy [6].

In this study, we introduce the use of a combination of micronutrients, each of which has previously been shown individually in the medical and scientific literature to benefit BMD outcomes. To assess the value of any therapy for compromised bone strength, one should ask if it fulfills the following criteria:

- protection from fragility fractures at multiple skeletal sites;
- rapid onset of action in order to provide benefit as soon as possible;
- minimal side effects for maximum tolerability;
- long-term safety;
- patient acceptability.

It appears that the COMB strategy may fulfill many of these criteria. The protection from fragility fractures was suggested by the occurrence of no fractures in the group taking the intervention as well as a notable increase in BMD at femur, hip, and spine sites on the BMD testing. A major proportion of the patients had an increase in BMD of more than 3% within the first year of therapy alone. There were no reported side effects with the use of this therapy among those taking the intervention for the year and the literature suggests long-term safety with each of these agents—this might contribute to greater compliance with the subgroup of patients who are reluctant to use pharmacologic therapies. For those who completed the course of therapy, the acceptability was high.

In response to these findings, two questions arise:

- How does nutritional supplementation work for disordered bone strength?
- Does micronutrient therapy have any role in mainstream medical practice?

A scientific approach to illness necessitates exploring the source etiology of health problems when possible and addressing causative determinants, including biochemical deficiencies [75]. From the results of this study it is hypothesized that osteoporosis in some cases may be related to nutritional deficiency of selected nutrients. Nutrient biochemicals are the fundamental building blocks of the human body, including the skeletal system; deficiency of required nutrients results in disordered biology and disease. Repletion of such nutrients may spontaneously correct and perhaps cure bone compromise in both young and mature patients. Just as restoring gestational folic acid to prevent open neural tube defects or supplementing with iron to ameliorate iron-deficiency anemia are recognized as credible and indicated nutritional interventions, remediation of essential biochemicals to restore and maintain bone strength is both evidence-based and science-based medicine. Further research of micronutrient strategies with longer followup will be needed to explore the effectiveness of this approach to disorders of bone health, but these preliminary results are encouraging indeed.

## Figures and Tables

**Table 1:** Combination of micronutrients (COMB) Protocol for Bone Health.

COMB protocol for bone health	
(1) Docosahexanoic acid or DHA (from Purified Fish Oil): 250 mg/day	
(2) Vitamin D_3_: 2000 IU/day	
(3) Vitamin K_2_ (non-synthetic MK_7_ form): 100 ug/day	
(4) Strontium citrate: 680 mg/day	
(5) Elemental magnesium: 25 mg/day	
(6) Dietary sources of calcium recommended	
(7) Daily impact exercising encouraged	

**Table 2:** Distribution of bone density diagnosis in the sample.

	Total (77)	Females (72)	Males (5)
Postmenopausal	—	58 (81%)	—
Normal BMD	19 (25%)	16 (22%)	3 (60%)
Reduced BMD	4 (5%)	3 (4%)	1 (20%)
Osteopenia	32 (42%)	32 (44%)	0
Osteoporosis	22 (29%)	21 (29%)	1 (20%)

**Table 3:** Pre and Posttreatment bone density.

	Pretreatment result (Mean ± SD)	Posttreatment result (Mean ± SD)	*P* value
Femoral neck (z-score)	−0.51 ± 0.74	−0.24 ± 0.81	0.03*
Total hip (z-score)	−0.27 ± 0.82	−0.06 ± 0.84	0.12
Lowest hip site (z-score)	−0.61 ± 0.71	−0.27 ± 0.81	0.006*
L1–L4 spine (z-score)	−0.85 ± 0.98	−0.39 ± 1.07	0.006*
Lowest spine site (z-score)	−1.40 ± 0.95	−0.67 ± 1.07	<0.001*

*Significant value.

**Table 4:** One year of therapy with the COMB protocol compared to strontium ranelate and bisphosphonate medications.

Percent change	COMB protocol: one year whole group (postmenopausal females)	Comparison to Strontium Ranelate at one year [21]	Comparison to Alendronate at one year [44]	Comparison to Risedronate at one year [45]
Femoral neck	4%	2%	2%	2%
Total hip	3%	3-4%	2%	Not calculated
Lowest hip site	4%	Not calculated	Not calculated	Not calculated
Total spine	6%	5-6%	4%	4%
Lowest spine site	8%	Not calculated	Not calculated	Not calculated

**Table 5:** Comparison of outcomes between patients who commenced the COMB protocol for declined drug therapy and those who previously showed no improvement on bisphosphonates.

Percent change	Patients who declined therapy with bisphosphonate (*N* = 48)	Patients who reported failure with previous bisphosphonate therapy (*N* = 29)
Femoral neck	3%	5%
Total hip	3%	3%
Lowest hip site	4%	5%
Total spine	6%	6%
Lowest spine site	9%	8%
